# Successful treatment of G-CSF-related aortitis with prednisolone during preoperative chemotherapy for breast cancer: a case report

**DOI:** 10.1186/s40792-021-01111-z

**Published:** 2021-01-14

**Authors:** Yoichi Koyama, Kayo Adachi, Mio Yagi, Yoko Go, Kyoko Orimoto, Saori Kawai, Natsuki Uenaka, Miki Okazaki, Mariko Asaoka, Saeko Teraoka, Ai Ueda, Kana Miyahara, Takahiko Kawate, Hiroshi Kaise, Kimito Yamada, Takashi Ishikawa

**Affiliations:** Department of Breast Oncology and Surgery, Tokyo Medical University, 6-1-1 Nishishinjuku, Shinjuku-ku, Tokyo 160-8402 Japan

**Keywords:** Aortitis, Breast cancer, Chemotherapy, Granulocyte colony-stimulating factor, Steroid

## Abstract

**Background:**

With the introduction of dose-dense therapy, the use of primary pegfilgrastim (PEG-G) has been increasing in breast cancer treatment. A rare side effect of PEG-G is aortitis. We describe a case of PEG-G-induced aortitis.

**Case presentation:**

The patient was a 43-year-old woman with stage IIA breast cancer. Due to the subtype of triple-negative breast cancer, preoperative dose-dense epirubicin–cyclophosphamide chemotherapy was started. PEG-G was administered on day 3 after the first cycle of epirubicin–cyclophosphamide chemotherapy. On day 11, she had a fever (39.4 °C) and an elevated C-reactive protein level (27.1 mg/dL). Emergency computed tomography revealed diffused wall thickening of the aortic arch without any other signs of infection. Despite administering antibiotics, her general condition and laboratory findings deteriorated until day 18. Based on these observations, she was diagnosed with PEG-G-induced aortitis. Antibiotics were discontinued, and she was treated with prednisolone thereafter. Subsequently, her clinical symptoms and laboratory findings improved around day 39. A second computed tomography scan revealed a decrease in the aortic arch wall thickening, and she was discharged on day 43.

**Conclusions:**

We successfully treated PEG-G-induced aortitis using prednisolone. Although this side effect is rare, cancer patients receiving PEG-G for chemotherapy should be monitored for aortic inflammation.

## Background

Granulocyte colony-stimulating factor (G-CSF) is used as a standard treatment for chemotherapy-related neutropenia in patients with blood and solid tumors. In Japan, filgrastim/lenograstim was approved in 1991 to treat neutropenia, and pegfilgrastim (PEG-G) was approved in 2014 to prevent neutropenia. G-CSF acts on the granulocyte lineage, stimulating neutrophil recruitment from the bone marrow to the peripheral blood, thereby promoting differentiation and maturation of these cells. Primary prophylaxis with PEG-G is warranted for patients undergoing intensive chemotherapy regimens or for those who are at greater risk due to age or comorbidities. Dose-dense therapy has been introduced to patients with advanced and/or aggressive disease, such as triple-negative breast cancer. Thus, the use of primary PEG-G has been increasing accordingly.

The main side-effects of PEG-G are musculoskeletal pain, injection site reaction, and liver dysfunction; anaphylaxis, interstitial lung disease, acute respiratory distress syndrome, splenomegaly, blast increase, capillary leak syndrome, and skin/large vasculitis have been reported as rare but serious side-effects [1]. G-CSF preparations are one of the typical drugs reported to cause drug-related vasculitis [2, 3] and are considered to be one of the risk factors for drug-induced vasculitis [4].

Here, we report a rare case of PEG-G-induced aortic inflammation in a patient receiving preoperative chemotherapy for breast cancer.

## Case presentation

A 43-year-old woman presented to our hospital with a right breast tumor. Core needle biopsy revealed invasive ductal carcinoma, which was estrogen and progesterone receptor-negative, HER2-negative, and Ki-67-positive (80%); she was diagnosed with stage IIA right-sided breast cancer. She was asymptomatic with no fever and had no significant medical or family history. Laboratory tests showed inflammation (white blood cells [WBC]: 10,800/μL and C-reactive protein [CRP]: 6.48 mg/dL). There were no other abnormal findings**,** or derangements in liver and kidney function (Table 1). Based on the profile of triple-negative breast cancer, dose-dense epirubicin–cyclophosphamide (EC) (epirubicin 90 mg/m^2^ day 1 + cyclophosphamide 600 mg/m^2^ day 1 bi-weekly × 4 cycles) followed by paclitaxel (175 mg/m^2^ on day 1 bi-weekly × 4 cycles) was planned as preoperative chemotherapy.

**Table 1:** Laboratory findings on the first visit

*Blood cell count*		*Biochemistry*	
White blood cells (/µL)	10,800	AST (U/L)	19
Neutrophils (%)	71.9	ALT (U/L)	19
Eosinophils (%)	1.7	LDH (U/L)	201
Basophil (%)	0.7	Total bilirubin (mg/dL)	0.31
Lymphocytes (%)	5.6	ALP (U/L)	201
Red blood cells (/µL)	5.01 × 10^6^	Total protein (g/dL)	7.6
Hemoglobin (g/dL)	12.9	Albumin (g/dL)	-
Platelet count (/µL)	328 × 10^6^	Creatine kinase (U/L)	105
*Coagulation*		BUN (mg/dL)	8.1
Prothrombin time (%)	11.6	Creatinine (mg/dL)	0.69
PT-INR	0.95	Na (mmol/L)	138
APTT (second)	31.3	K (mmol/L)	4.4
D-dimer (µg/dL)	–	Cl (mmol/L)	104
Antithrombin III (%)	–	CRP (mg/dL)	6.48

*PT-INR* international normalized ratio of prothrombin time, *APTT* activated partial thromboplastin time, *AST* aspartate aminotransferase, *ALT* alanine aminotransferase, *LDH* lactate dehydrogenase, *ALP* alkaline phosphatase, *BUN* blood urea nitrogen, *CRP* C-reactive protein

**Fig. 1:**
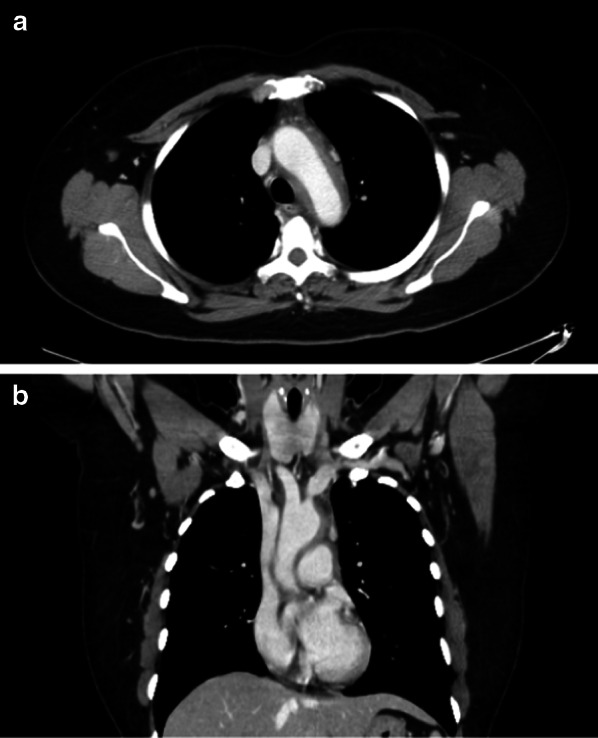
Computed tomography (CT) scan on admission. CT scan on day 11 reveals diffused wall thickening centering on the aortic arch. Vasculitis was suspected of causing the fever. **a** Axial plane; **b** coronal plane

PEG-G was administered 3 days after the first EC treatment. On day 8, the patient developed a fever of 38.3 °C, and was admitted on day 11 due to lingering fever. On admission, her body temperature was 39.4 °C; the laboratory data showed: leukocytosis (WBC: 28,700/µL), elevation of CRP (27.1 mg/dL), prothrombin time/activated partial thromboplastin time (PT/APTT) prolongation (14.6%/46.8 s), elevation of D-dimer level (2.83 µg/mL), and liver dysfunction (aspartate aminotransferase, 356 U/L; alanine aminotransferase, 536 U/L). Immunological tests revealed a 40-fold lower level of anti-nuclear antibodies; myeloperoxidase anti-neutrophil cytoplasmic antibody and proteinase 3 anti-neutrophil cytoplasmic antibody tests were negative. Her immunoglobulin G4 level was normal, and she tested negative for mumps virus, mycobacterium tuberculosis, primary biliary cirrhosis, Epstein–Barr virus, and cytomegalovirus infection. Urinalysis showed no abnormal findings. The blood and urine cultures were negative (Table 2). A CT scan revealed diffused wall thickening centered on the aortic arch, suggesting vasculitis (Fig. 1). Carotid echocardiography showed no clear signs of inflammation.

**Table 2:** Laboratory findings on admission

*Blood cell count*		IgG (mg/dL)	672
White blood cells (/µL)	28,700	IgA (mg/dL)	172
Neutrophils (%)	90.1	IgM (mg/dL)	9
Eosinophils (%)	0	IgG4 (mg/dL)	43
Basophil (%)	0.1	ANA	Negative
Lymphocytes (%)	3.7	MPO-ANCA	Negative
Red blood cells (/µL)	4.37 × 10^6^	PR3-ANCA	Negative
Hemoglobin (g/dL)	11.3	Mumps IgG	Negative
Platelet count (/µL)	328 × 10^6^	Mumps IgM	Negative
*Coagulation*		Tb IFN-γ	Negative
Prothrombin time (%)	14.6	AMA	Negative
PT-INR	1.21	EBEA IgG	Negative
APTT (second)	46.8	EBNA IgG	Negative
D-dimer (µg/dL)	2.83	CMV VC7HRP	Negative
Antithrombin III (%)	75	*Serology*	
*Biochemistry*		TPHA	Negative
AST (U/L)	356	RPR	Negative
ALT (U/L)	536	HBs ag	Negative
LDH (U/L)	822	HBs ab	Negative
Total bilirubin (mg/dL)	0.38	HCV ab	Negative
ALP (U/L)	713	Blood culture	Negative
Total protein (g/dL)	5.4	Urine culture	Negative
Albumin (g/dL)	2.3	*Urinalysis*	
Creatine kinase (U/L)	38	Specific gravity	1.005
BUN (mg/dL)	10.3	pH	5.5
Creatinine (mg/dL)	0.94	Red blood counts	Negative
Na (mmol/L)	137	White blood counts	Negative
K (mmol/L)	4.4	Nitrous acid	Negative
Cl (mmol/L)	103	Color yellow	Negative
CRP (mg/dL)	27.1		

*PT-INR* international normalized ratio of prothrombin time, *APTT* activated partial thromboplastin time, *AST* aspartate aminotransferase, *ALT* alanine aminotransferase, *LDH* lactate dehydrogenase, *ALP* alkaline phosphatase, *BUN* blood urea nitrogen, *CRP* C-reactive protein, *IgG* immunoglobulin G, *IgA* immunoglobulin A, *IgM* immunoglobulin M, *IgG4* immunoglobulin G4, *ANA* anti-nuclear antibody, *MPO-ANCA* myeloperoxidase anti-neutrophil cytoplasmic antibody, *PR3-ANCA* proteinase 3 anti-neutrophil cytoplasmic antibody, *Tb IFN-γ* tuberculosis interferon-γ, *AMA* anti-mitochondrial antibody, *EBEA IgG* Epstein–Barr virus early antigen immunoglobulin G, *EBVA IgG* Epstein–Barr virus nuclear antigen immunoglobulin G, *CMV C7HRP* cytomegalovirus antigen C7HRP, *TPHA* *Treponema pallidum* hemagglutination test, *RPR* rapid plasma reagin, *HBs ag* hepatitis B surface antigen, *HBs ab* hepatitis B surface antibody, *HCV ab* hepatitis C virus antibody

**Fig. 2:**
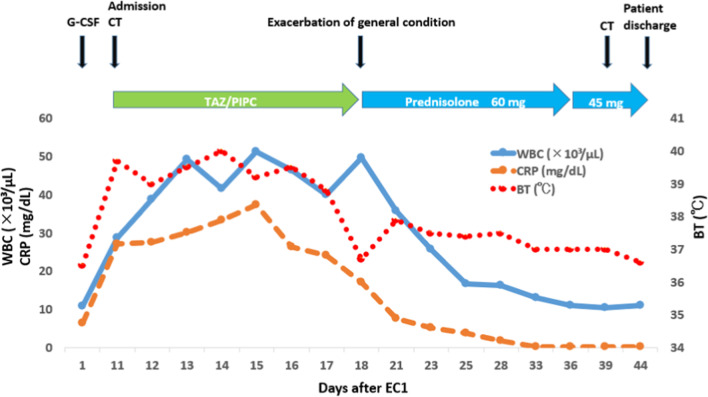
The clinical course of the case. Pegfilgrastim (PEG-G) is administered on day 3. PEG-G-related aortitis is suspected based on the CT scan on day 11; steroid treatment is initiated on day 18, after which the patient’s general condition and laboratory findings improve dramatically. *G-CSF* granulocyte colony-stimulating factor; *WBC* white blood cells; *CRP* C-reactive protein; *CT* computed tomography; *BT* body temperature; *TAZ/PIPC* tazobactam/piperacillin; *EC1* the first cycle of epirubicin–cyclophosphamide

**Fig. 3:**
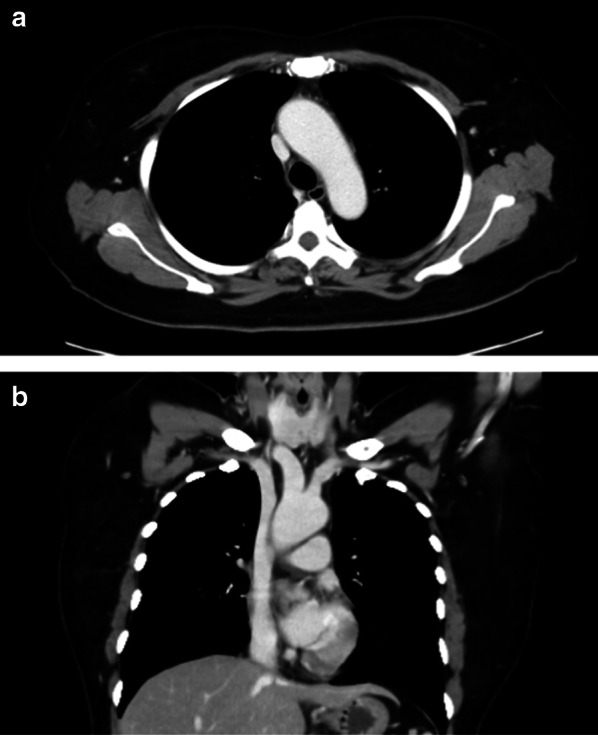
Computed tomography (CT) scan after steroid treatment. CT scan on day 39 showing decrease in the wall thickening of the aortic arch. **a** Axial plane; **b** coronal plane

Although the bacterial cultures were negative, she was treated with antibiotics (tazobactam/piperacillin 4.5 g, four times a day) commencing on day 11. However, they were discontinued on day 18 due to the deterioration of her general condition. PEG-G-induced aortitis was suspected based on the CT scan and the ineffectiveness of antibiotics. She was then treated with 60 mg of high-dose prednisolone (1.0 mg/kg/day), which led to a rapid improvement in her general condition and laboratory findings. The CRP levels were within the normal range (< 0.30 mg/dL) on day 36**,** and the dose of prednisolone was reduced to 45 mg/day (Fig. 2). On day 39, the wall thickening of the aortic arch decreased (Fig. 3), and she was discharged on day 43.

Two months after steroid treatment, the patient underwent breast-conserving surgery with sentinel lymph node biopsy. Her postoperative treatment consisted of chemotherapy followed by irradiation (42.5 Gy/16 Fr; performed 3 months after steroid treatment). Chemotherapy with EC can potentially cause hepatic and renal disorders, and taxane anticancer drugs such as docetaxel and paclitaxel, which are the standard treatment, may cause myelosuppression requiring G-CSF use. Additionally, postoperative oral capecitabine has been reported to improve prognosis [5]. Therefore, capecitabine was started 4 months after steroid treatment (at a dose of 1000 mg/m^2^ twice a day for days 1–14 of a 21-day cycle; 8 courses were planned in total). The patient is currently undergoing follow-up with no treatment, and the breast cancer has not recurred.

During steroid treatment, prednisolone was administered orally with doses gradually decreasing from 30 mg after discharge; 15, 12, and 10 mg were administered during surgery, irradiation, and anticancer drug therapy, respectively. Prednisolone is being continued 1 year after the onset, at a dose of 5 mg, and there has been no relapse of vasculitis.

## Conclusion

The patient described was diagnosed with PEG-G-related aortitis based on the presence of fever, posterior cervical and chest pain, and CT findings of the aortic arch 8 days after PEG-G administration. The clinical course and effectiveness of steroids, and the ineffectiveness of antibiotics also indicated a non-bacterial source of inflammation.

Vasculitis with no specific symptoms develops in various pathological conditions such as syphilis, collagen disease, and the paraneoplastic syndrome of malignant lymphoma. It also occurs in response to various drugs, including antibiotics such as cefaclor and erythromycin, non-steroidal anti-inflammatory drugs such as indomethacin, anticancer drugs such as cisplatin and fluorouracil, and biological drugs such as G-CSF and interferon-α [2, 3, 6, 7].

The pathophysiology of drug-induced vasculitis is diverse, including the involvement of anti-neutrophil cytoplasmic antibodies and immune complexes, damage to vascular endothelial cells, and activation of drug-specific T cells. G-CSF is a biological drug, whose use has been increasing rapidly in recent years; it promotes differentiation and growth of neutrophils and simultaneously induces the release of inflammatory cytokines such as interleukin-6 and tumor necrosis factor-α. Since it causes arteriosclerosis, aneurysms, and arteritis, the cornerstone of the pathological condition is considered to be the activation of neutrophils [8].

Aortitis is more common in cancer patients receiving G-CSF along with chemotherapy; however, no specific regimen or type is known to induce it. It is also more common in women than in men.

G-CSF-associated aortitis was first reported in 2004 [9], with 57 cases from the United States. Most of them included patients with breast cancer and hematological malignancies, and the suspected causative drugs were filgrastim in 21 cases and PEG-G in 33 cases [10]. A study from Japan by Oshima et al. [11] evaluating the association between G-CSF administered with chemotherapy and aortitis reported 25 cases of aortitis among the 102,014 cases of malignant neoplasms evaluated. G-CSF preparations were used in the 3409 cases, and the 16 cases (0.47%; 11, 4, 3 cases of PEG-G, filgrastim, lenograstim) developed aortitis. Among the 25 cases with aortitis, 22 (88%) were women. The median age of the 16 cases with G-CSF-related aortitis was 56.9 (± 10.4) years, and 15 of them (94%) were women. The 4 cases of G-CSF-related aortitis showed a similarity to Takayasu arteritis in terms of the clinical characteristics. Aortitis significantly correlated with G-CSF administration (odds ratio [OR] 45.87, *P* < 0.001), male sex (OR 0.13, *P* = 0.001), breast cancer (4 cases) (OR 24.71, *P* = 0.000), malignant lymphoma (3 cases) (OR infinity, *P* = 0.002), and ovarian cancer (4 cases) (OR 19.70, *P* = 0.001) [10].

Furthermore, the patient had a high CRP level before treatment was started. However, there were no abnormal findings of liver and kidney function, no symptoms such as fever or fatigue, and no relevant medical history. Although further examinations needed to be performed, she was in a good general condition and wanted to start treatment for an aggressive disease as soon as possible. Neoadjuvant chemotherapy was chosen as the preferred treatment and was subsequently started. When the episode occurred, we discussed this case with a multidisciplinary team including clinical oncologists and internists specialized in collagen disease. Based on radiological and laboratory findings, we concluded that PEG-G caused this symptom. However, it is still possible that she might have had an underlying inflammatory condition, such as Takayasu arteritis.

It has been reported that chronic inflammatory autoimmune diseases such as systematic lupus erythematosus, Felty’s syndrome, psoriatic arthritis, and multiple sclerosis may also induce systemic vasculitis by complement system activation and cytokine and chemokine production due to the activation of neutrophils after G-CSF administration [12–14]. Therefore, G-CSF administration should be carefully monitored in patients with systemic inflammatory diseases. In our patient, the symptoms and the swollen aortic arch decreased soon after steroid administration. Five months after the steroid treatment, a CT scan showed that the thickening of the aortic wall had disappeared entirely, and the CRP level, which was higher at the first visit, was within the normal range.

Although no apparent risk factors have been reported, the present case highlights the risk of aortitis in patients who receive G-CSF therapy. G-CSF-related aortitis is rare, but physicians should be aware of it when treating cancer patients.

Moreover, PEG-G-induced aortic inflammation is also rare; however, given its frequent use for chemotherapy, its administration should be carefully monitored to avoid aortitis.

## Data Availability

All data supporting this article are included in this manuscript.

## Abbreviations

CRP: C-reactive protein
CT: Computed tomography
EC: Epirubicin–cyclophosphamide
G-CSF: Granulocyte colony-stimulating factor
OR: Odds ratio
PEG-G: Pegfilgrastim
PT/APTT: Prothrombin time/activated partial thromboplastin time
WBC: White blood cells
